# High Prevalence and Diversity Characteristics of *bla*_NDM_, *mcr*, and *bla*_ESBLs_ Harboring Multidrug-Resistant *Escherichia coli* From Chicken, Pig, and Cattle in China

**DOI:** 10.3389/fcimb.2021.755545

**Published:** 2022-02-07

**Authors:** Zhihai Liu, Ke Wang, Yaru Zhang, Lining Xia, Li Zhao, Changmei Guo, Xudong Liu, Liting Qin, Zhihui Hao

**Affiliations:** ^1^ College of Chemistry and Pharmaceutical Sciences, Qingdao Agricultural University, Qingdao, China; ^2^ College of Veterinary Medicine, China Agricultural University, Beijing, China; ^3^ Department of Microbiology and Immunology, College of Husbandry and Veterinary Medicine, Henan Agricultural University, Zhengzhou, China; ^4^ College of Veterinary Medicine, Xinjiang Agricultural University, Wulumuqi, China; ^5^ Department of Instruments, Autobio Labtec Instruments Co., Ltd, Zhengzhou, China; ^6^ Academy of Poultry Industry Research, The New Hope Liuhe Co., Ltd., Qingdao, China; ^7^ College of Veterinary Medicine, South China Agricultural University, Guangzhou, China

**Keywords:** multidrug resistance, food animal, ESBL, *Escherichia coli*, NDM-5

## Abstract

The objective of this study was to understand the diversity characteristics of ESBL-producing *Escherichia coli* (ESBL-EC) in chicken, pig, and cattle. A high prevalence of ESBL-EC (260/344) was observed in all food animals with prevalence rates of 78.6% (110/140) for chicken, 70.7% (58/82) for cattle, and 75.4% (92/122) for swine. However, the resistance rates presented significant differences in different animal origin ESBL-EC, where resistance to CTX, GEN, IMP, NEO, and OFL was the highest in chicken ESBL-EC, then in cattle, and the lowest in swine. Seriously, most ESBL-EC harbor multidrug resistance to antibiotics (MDR, ≥3 antibiotic categories), and the MDR rates of ESBL-EC were the highest in chicken (98.18%), followed by swine (93.48%), and the lowest in cow (58.62%), while the same trend also was observed in MDR of ≥5 antibiotic categories. This high prevalence and resistance can be partly interpreted by the high carriage rates of the β-lactamases CTX-M (*n* = 89), OXA (*n* = 59), SHV (*n* = 7), and TEM (*n* = 259). A significant difference of β-lactamase genes also presented in different animal species isolates, where the chicken origin ESBL-EC possessed higher carriage rates of almost all genes tested than cattle and swine. Notably, eight chicken origin ESBL-EC carried transferable plasmid-mediated *bla*_NDM-1_ or *bla*_NDM-5_, especially, of which four ESBL-EC also contained the colistin resistance gene *mcr-1*, as confirmed by genomic analysis. More interestingly, two deletion events with a 500-bp deletion in ΔIS*Aba125* and a 180-bp deletion in *dsbC* were observed in three *bla*_NDM-5_ IncX3 plasmids, which, as far as we know, is the first discovery. This showed the instability and horizontal transfer of *bla*_NDM_ genetic context, suggesting that *bla*_NDM_ is evolving to “pack light” to facilitate rapid and stable horizontal transfer. Sequence types (STs) and PFGE showed diversity patterns. The most prevalent STs were ST48 (*n* = 5), ST189 (*n* = 5), ST206 (*n* = 4), ST6396 (*n* = 3), ST10 (*n* = 3), and ST155 (*n* = 3), where ST48 ESBL-EC originated from three food animal species. The STs of all *bla*_NDM_-positive ESBL-EC were attributed to three STs, namely, ST6396 (*n* = 2), ST206 (*n* = 2), and ST189 (*n* = 4), where ST189 was also the unique type for four *mcr-1*-carrying ESBL-EC. In conclusion, we suggest that the three animal species ESBL-EC show similar high prevalence, diversity in isolate lineages, and significant discrepancies in antibiotic resistance and resistance genes. This suggests that monitoring and anti-infection of different food animal origin ESBL-EC need different designs, which deserves more attention and further surveillance.

## Introduction

Antimicrobial resistance (AMR) is a serious threat to public health. The annual human deaths will rise from the current 0.7 million to 10 million with an estimated $100 trillion in economic losses by 2050 due to AMR (O’Neill, 2016; Friedrich, 2019). The extensive use of antimicrobials in humans, animals, and the environment has generated populations of extended-spectrum β-lactamase (ESBL)-producing Enterobacteriaceae, carbapenem-resistant Enterobacteriaceae (CRE), vancomycin-resistant enterococci (VRE), methicillin-resistant *Staphylococcus aureus* (MRSA), and so on across the globe (Okpara et al., 2018), in which ESBL-producing Enterobacteriaceae and CRE, such as NDM-producing Enterobacteriaceae, are the most prevalent in animals (Wang et al., 2017). Animals, especially food animals, are not only the reservoirs of antibiotic resistance genes (ARGs) and antibiotic resistance bacteria (ARB) but also the generators of novel resistance mechanisms and genes. Recently, novel mechanisms and genes were first revealed in food animal bacteria, such as mobile colistin resistance (*mcr-1*) in pig (Liu et al., 2016) and plasmid-mediated tigecycline resistance gene *tet*(X) in pig and chicken (He et al., 2019; Sun et al., 2019). In our previous work, a series of novel variant genes were also firstly reported in food animal origin isolates, such as novel carbapenemase-encoding genes *bla*_NDM-17_ (Liu et al., 2017), *bla*_NDM-20_ (Liu et al., 2018), and *bla*_VIM-48_ (Liu et al., 2019); novel *mcr* variants *mcr-3* (Yin et al., 2017); and plasmid-mediated high-level tigecycline resistance genes *tet*(X3) and *tet*(X4) (He et al., 2019). Currently, the important ARGs in animals are *bla*, *mcr*, *cfr*, and *tet* genes and their variants (de Alcântara Rodrigues et al., 2020; Ling et al., 2020), where *bla* (*bla*_KPC_, *bla*_NDM_, *bla*_VIM_, and *bla*_OXA-48/23_), *mcr*, and *tet* gene-mediated AMR are closely associated to the last resort antibiotics in the human clinic (Guerra et al., 2014). Worryingly, all those ARB and ARGs can transfer to human through the consumption of products of animal origin (de Alcântara Rodrigues et al., 2020). Accordingly, AMR and ARGs in food animals need more attention.

Among numerous resistance mechanisms, the expression of ESBL is the most prevalent and increasingly common and has become the basic resistance mechanism that persists over other ARGs (Bush and Bradford, 2020). For example, *bla*_ESBLs_ genes were observed in almost all CRE or *mcr*-positive pathogens (Bush and Bradford, 2020; Ling et al., 2020). ESBLs can hydrolyze β-lactam antibiotics including the penicillins and cephalosporins, which is still a common clinical difficulty in fighting infection. The most prevalent and typical ESBLs are the *bla*_TEM_, *bla*_SHV_, and *bla*_CTX-M_ genes which have evolved into dozens of subtypes with increasing enzyme activities by amino acid substitutions. These β-lactamases have been isolated from humans (Pitout and Laupland, 2008; Musicha et al., 2017; Wittekamp et al., 2018), the environment (Runcharoen et al., 2017), and animals in many countries including China (White et al., 2001) and represent an emerging general public health threat. In particular, animals and farm environments are important reservoirs of ESBL-producing bacteria (Seiffert et al., 2013; Kim et al., 2019). A high prevalence of ESBL producers along with a high level of diversity in ESBL genes has been reported in broilers and poultry production chains (Dame-Korevaar et al., 2019). In addition, animal wastes carrying ESBLs lead to biological contamination and accumulation in animal food, vegetables, water, and soil (Caltagirone et al., 2017; Dorado-García et al., 2018; Sen and Sarkar, 2018). So, ESBL genes can spread from animals to humans and can be directly transmitted to farmers (Kola et al., 2012; Dohmen et al., 2015). Seriously, the coexistence of other resistance genes, such as *fosA*, *sul*, *bla*_NDM_, and *mcr*, is frequent in ESBL-producing pathogens, resulting in multidrug resistance (Wang et al., 2017). Consequently, this will compromise the effectiveness of β-lactams for the treatment of disease in both people and animals, which will ultimately aggravate financial burden on society.

Antimicrobial resistance is derived from antibiotic selection and exposure, and dissemination of ESBL-producing bacteria is associated with the heavy use of cephalosporins (Allcock et al., 2017). Globally, 63,151 tons of antibiotics (all classes) were used to treat livestock in 2010, and it is predicted to reach 105,596 tons by 2030 (Van Boeckel et al., 2015). Given the global estimates of antimicrobial consumption based on species-specific coefficients of antimicrobial consumption per population correction unit (PCU), cattle, chicken, and pig are the primary consumers of antibiotics and the highest use is reported in pig (Van Boeckel et al., 2015), where a significant difference in antimicrobial consumption is estimated which is generally lower (45 mg/PCU) for cattle than for chicken (148 mg/PCU) and pig (172 mg/PCU). Studies have shown that resistance correlates with antibiotic consumption (Mather et al., 2012). Accordingly, the resistance patterns should follow the usage pattern. Withal, the discrepancy in their physiology, species, and growth environment may also affect AMR. All those may influence the prevalence of drug-resistant bacteria.

So, in China, as the largest consumer of veterinary drugs, in which most are β-lactams used to treat livestock (Van Boeckel et al., 2015), a comprehensive study is necessary to better understand the prevalence characteristics of ESBL-producing *Escherichia coli* (ESBL-EC) in animals. The aim of this study was to characterize the diversity characteristics of ESBL-producing *E. coli* and the prevalence characteristics and the prevalence of *bla*_NDM_, *mcr*, and *bla*_ESBLs_ from the different food animals, such as chicken, pig, and cattle.

## Materials and Methods

### Sample Collection and Bacterial Strain Identification

The fecal samples (*n* = 344) in this study were collected from livestock farms in Guangdong, Shandong, Xinjiang, and Heilongjiang provinces during 2015–2019. Samples were taken from chicken, swine, and cattle feces. These farms had records of β-lactam antibiotic usage for preventing and treating bacterial infections (data not shown). Immediately upon receipt of samples by the laboratory, they were plated on MacConkey agar plates (Luqiao, Beijing, China) and incubated at 37°C for 16–18 h. Red colonies were selected and enriched by cultivation in 2 ml Mueller–Hinton broth (Luqiao) and plated on methylene blue agar (Luqiao) plates containing 2 µg/ml of cefotaxime. Presumptive ESBL-producing *E. coli* were collected and genomic DNA was extracted using a Fast Pure Bacteria DNA Isolation Mini Kit (Vazyme Biotech, Nanjing, China). Bacterial species were confirmed using 16S rDNA PCR amplification and sequencing as previously described (Kim et al., 2010).

### Antimicrobial Susceptibility Testing

The broth microdilution method was used to determine the minimal inhibitory concentrations (MIC) for the following antibiotic categories: i) carbapenems: imipenem (IMP); ii) penicillins: amoxicillin (AMX); iii) cephalosporins: cephalexin (CN), cefotaxime (CTX), and cefepime (FEP); iv) tetracyclines: tetracycline (TET) and tigecycline (TGC); v) aminoglycosides: gentamicin (GEN) and neomycin (NEO); vi) aminocyclitol: spectinomycin (SPT); vii) colistin (CST); and viii) quinolones: ofloxacin (OFL) (Hubei Widely Chemical Technology Co., Ltd.). *Escherichia coli* ATCC 25922 was used as the quality control strain in MIC testing. All procedures and test interpretations followed the Clinical and Laboratory Standards Institute (CLSI) guidelines (CLSI, 2021). Multidrug-resistant (MDR) isolates were defined as possessing resistance to three or more antibiotic categories (Magiorakos et al., 2012).

### Identification of Resistance Genes

All presumptive ESBL-producing isolates were screened by PCR for the β-lactamase genes *bla*_SHV_, *bla*_TEM_, and *bla*_CTX-M_ and carbapenemase-encoding genes *bla*_NDM_, *bla*_IMP_, *bla*_KPC_, *bla*_VIM_, *bla*_OXA_, *bla*_AIM_, *bla*_BIC_, *bla*_DIM_, *bla*_GIM_, *bla*_SIM_, and *bla*_SPM_ (Poirel et al., 2011; Casella et al., 2018), and each sample was tested at least twice. The genes *bla*_CTX-M_, *bla*_NDM_, and *bla*_OXA_ were subtyped by PCR and sequencing as previously described (Poirel et al., 2011), and *bla*_NDM_ subtypes were further confirmed by whole genome sequencing (WGS). PCR primers and conditions for PCR amplification are listed in **Table 1**. PCR amplicons were separated by electrophoresis through 1.5% agarose gels, stained with EtBr, and visualized under UV light. Amplicons were sequenced to confirm identity and analyzed using the BLAST algorithm (http://www.ncbi.nlm.nih.gov/blast/). *Escherichia coli* CCD1 as a *bla*_NDM_-positive control in our previous report (Liu et al., 2018) and *E. coli* ATCC 25922, as the negative control, were used to assay the feasibility of the PCR test to exclude adverse factors.

**Table 1:** Primers used for detection and sequencing of target genes in *Escherichia coli* isolates.

Genes		Primers (5′–3′)	Annealing temperature	Reference
*bla*_OXA-2_ group	OXA-2-F	AAGAAACGCTACTCGCCTGC	58°C	Poirel et al. (2011); Casella et al. (2018)
	OXA-2-R	CCACTCAACCCATCCTACCC		
*bla*_CTX_	CTX-F	TCTTCCAGAATAAGGAATCCC	50°C	
	CTX-R	CCGTTTCCGCTATTACAAAC		
*bla*_SHV_	SHV-F	TGGTTATGCGTTATATTCGCC	61°C	
	SHV-R	GGTTAGCGTTGCCAGTGCT		
*bla*_OXA-10_ group	OXA-10-F	GTCTTTCGAGTACGGCATTA	53°C	
	OXA-10-R	ATTTTCTTAGCGGCAACTTAC		
*bla*_NDM_	NDM-F	CCAATATTATGCACTCTGTCGC	55°C	
	NDM-R	TCAGTGTAGCTTGTCTGCCATGT		
*bla*_TEM_	TEM-F	TCCGCTCATGAGACAATAACC	55°C	
	TEM-R	TTGGTCTGACAGTTACCAATGC		

### Pulsed Field Gel Electrophoresis and Multilocus Sequence Typing Analysis of ESBL-Positive *Escherichia coli*

The relatedness of ESBL-EC was assessed using pulsed field gel electrophoresis (PFGE) as previously described (Tenover et al., 1995). Here, 45 typical isolates were selected mainly based on source, resistance genes, and resistance (MIC). Normally, only one representative strain was selected among isolates possessing similar MIC or resistance genes, while the final sample size takes into account the number of different sources. They included swine (*n* = 10), cattle (*n* = 9), and chicken (*n* = 26). Briefly, agarose-embedded DNA was digested with *Xba*1 (Takara, Beijing, China) for 3 h at 37°C. DNA fragments were separated by electrophoresis in 0.5× TBE buffer at 14°C for 18 h using a CHEF-DR III electrophoresis system (Bio-Rad, Hercules, CA, USA) with pulse times of 2.2–54.2 s. *Escherichia coli* strain H9812 was used as standard for DNA size measurements. The gels were stained with EtBr and processed as above. PFGE results were analyzed using BioNumerics software v3.0 (Applied Maths Kortrijk, Belgium). All isolates successfully typed using PFGE were subjected to multilocus sequence typing (MLST) using the following loci: *recA*, *adk*, *fumC*, *icd*, *mdh*, *purA*, and *gyrB* (Kim et al., 2015). These PCR products were sequenced and the data were interpreted using the MLST database (http://enterobase.warwick.ac.uk/species/ecoli/allele_st_search) (Zhou et al., 2020). Cluster analysis of ST (sequence type) was performed using the BioNumerics software.

### Transconjugation *Assays* for *bla*_NDM_

Transferability of *bla*_NDM_ from animal *bla*_NDM_-positive *E. coli* isolates was assayed by conjugation using the azide-resistant strain *E. coli* J53 as the recipient (Liu et al., 2018). In brief, equal volumes of donor and recipient strains were mixed and filtered onto 0.45 µm filters that were then placed on Mueller–Hinton agar plates containing 100 µg/ml sodium azide and 1 µg/ml meropenem (Liu et al., 2019). At the same time, 10-fold dilutions were plated to determine transfer frequencies and presumptive transconjugants were screened using PCR (see above).

### Whole Genome Sequencing and Analysis for *bla*_NDM_-Positive *Escherichia coli*

The *bla*_NDM_-positive *E. coli* were further characterized by WGS using DNA extracted as described above (Wang et al., 2017). The NEXT Ultra DNA Library Prep kit (New England Biolabs, Beverly, MA, USA) was used to establish gene libraries of 150 bp with paired ends and sequenced using the Illumina HiSeq 2500 system at Bionova Biotech (Beijing, China). The SPAdes algorithm v.3.10.0 (http://cab.spbu.ru/software/spades/) was used to assemble raw data, and the assembled sequences were further analyzed using workflows obtained from the bacterial analysis pipeline (https://cge.cbs.dtu.dk/services/cge) in CGE (Center for Genomic Epidemiology) services.

## Results

### ESBL-Producing *Escherichia coli* and ARGs

In our study, we identified 260 ESBL-EC isolates from the 344 fecal samples, including 110 (78.6%, 110/140) from chicken, 58 (70.7%, 58/82) from cattle, and 92 (75.4%, 92/122) from swine. Our presumptive ESBL isolates were also examined for the three most common β-lactamase genes, in which the *bla*_TEM_ (99.62%, *n* = 259) was dominant and obviously higher than *bla*_SHV_ (2.69%, *n* = 7) and *bla*_CTX-M_ (34.2%, *n* = 89). Meanwhile, the carriage rate of *bla*_CTX-M_ in chicken was 80.9%, higher than that in swine (7.6%) and cattle (0%). Similarly, the carriage rate of *bla*_TEM_ and *bla*_SHV_ genes from chicken was higher than those from swine and cattle. Comparatively, the genes *bla*_TEM_, *bla*_SHV_, and *bla*_CTX-M_ from chicken were more prevalent than from swine and cattle. The carbapenemase-encoding genes in our sample population were represented by the *bla*_NDM_ gene, only found in chicken (*n* = 8), and by the *bla*_OXA_ gene in chicken, cattle, and swine at 33.6%, 25.9%, and 7.6%, respectively. Overall, *bla*_TEM-1_ was the predominant β-lactamase gene among the animal species screened in this study, and the *bla*_CTX-M-55_ gene was the major ESBL gene found in chicken.

Allele subtyping analysis indicated that 89 isolates harboring the *bla*_CTX-M_ gene were present in our ESBL-EC population with 7 known and 1 CTX-M-untypable. These were grouped as CTX-M-55 (77%), CTX-M-15 (5.6%), CTX-M-14 (4.45%), CTX-M-3 (4.45%), CTX-M-123 (1.1%), CTX-M-98 (2.2%), and CTX-M-65 (4.45%). We found only seven *bla*_CTX-M_-positive *E. coli* from swine. The CTX-M-55 variant was present in isolates from swine (*n* = 6) and chicken (*n* = 63), while only the CTX-M-55 and CTX-M-untypable subtypes were observed in swine.

**Figure 1:**
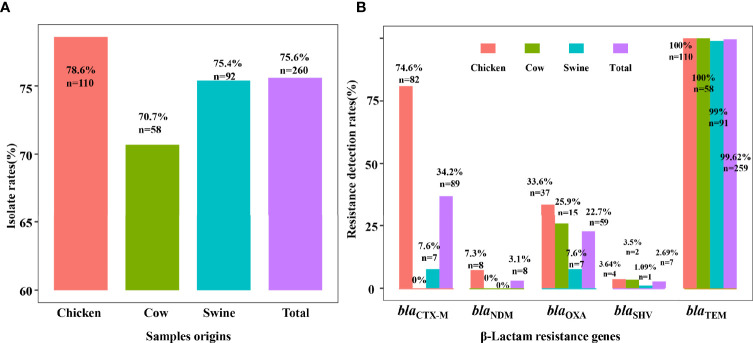
The prevalence of ESBL-EC and β-lactam resistance genes. Detection rates of ESBL-EC are shown in **(A)** and the carriage rates of β-lactam resistance genes are shown in **(B)**. The bars showed the detection rates of resistant isolates or carriage rates of resistance genes in ESBL-EC among different food animals. In total, 260 isolates were detected from 344 samples, including 110 from chicken (red), 58 from cow (green), and 92 from swine (blue).

When we examined the isolates from chicken, we found a diversity of β-lactamase. For example, the TEM subtype was TEM-1B, while the OXA series were represented by OXA-2 (*n* = 5) and OXA-10 (*n* = 54) variants. In the group of seven SHV subtypes, only one (SHV-12) was present and the others were untypable and tentatively named SHV-like. Importantly, eight isolates carried the *bla*_NDM_ gene with seven *bla*_NDM-5_ and a single *bla*_NDM-1_ isolate, in which four isolates also contained *bla*_NDM-5_ and *mcr-1.* All eight were collected from chicken. The other β-lactamase-related ARGs we examined for this study were absent in all isolates. In addition, ARG coexistence of resistance genes was common and OXA-10/TEM (*n* = 18), OXA-10/CTX-M/TEM (*n* = 32), and CTX-M/TEM (*n* = 48) were the primary groupings. The coexistence schemes of most genes were TEM/CTX-M/OXA-10/NDM (*n* = 4) and OXA-2/OXA-10/CTX-M/TEM (*n* = 1) (**Figure 1**).

### Antibiotic Resistance Profile

**Figure 2:**
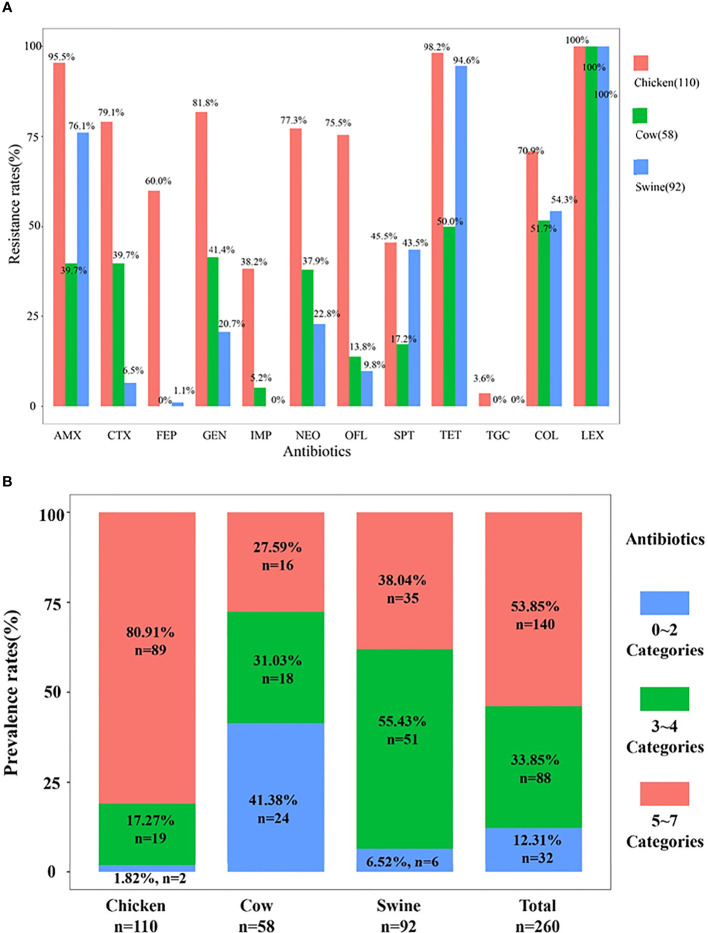
The resistance rates and multidrug resistance rates. The resistance rates of 260 isolates are shown in **(A)**. The prevalence rates of multidrug resistance among 260 ESBL-EC are shown in **(B)**.

Overall, the prevalence of drug-resistant isolates from chicken was higher than that from cattle and swine. All ESBL-EC were resistant to cephalexin and most were tigecycline susceptible. Resistance to CTX, GEN, IMP, NEO, and OFL was the highest in chicken, then in cattle, and the lowest in swine. Noticeably, more than 50% ESBL-EC from any animal origin were also resistant to colistin. Specifically, ESBL-EC from chicken exhibited diverse MDR patterns that included resistance to all tested antibiotics. The individual resistance rates for all antibiotics in isolates from chicken were at least 38% and the rates for seven antibiotics were >70% except for tigecycline (3.6%). The resistance rates for ESBL-EC from cattle were <51.7% (except for LEX). Similarly, ESBL-EC from swine also showed susceptibility to IMP and TGC and low resistance rates to FEP (1.1%) (**Figure 2A**).

The 12 antibiotics we tested in our study were from nine categories, and we found that MDR isolates were prevalent at rates of 87.69% (*n* = 228), where 88 isolates were resistant to 3–4 categories, while 140 isolates were resistant to 5–7 categories. MDR isolates from chicken and swine were the most prevalent (98.18%, *n* = 108; 93.48%, *n* = 84) and followed by cattle (>50%). The MDR isolates from chicken were primarily resistant to 5–7 antibiotic categories (81.9%, *n* = 89), and this rate was significantly higher than those for swine and cattle. Chicken also possessed a significant number of isolates that possessed resistance to 3–4 categories (17.27%, *n* = 19). In contrast, the cattle isolates displayed MDR rates to 3–4 and 5–7 categories that were almost equal. However, in swine MDR isolates, resistance to 3–4 categories (55.43%) greatly exceeded those in the 5–7 categories (**Figure 2B**). Therefore, different animal origin ESBL-EC demonstrated different MDR patterns.

### Analysis of PFGE and MLST

**Figure 3:**
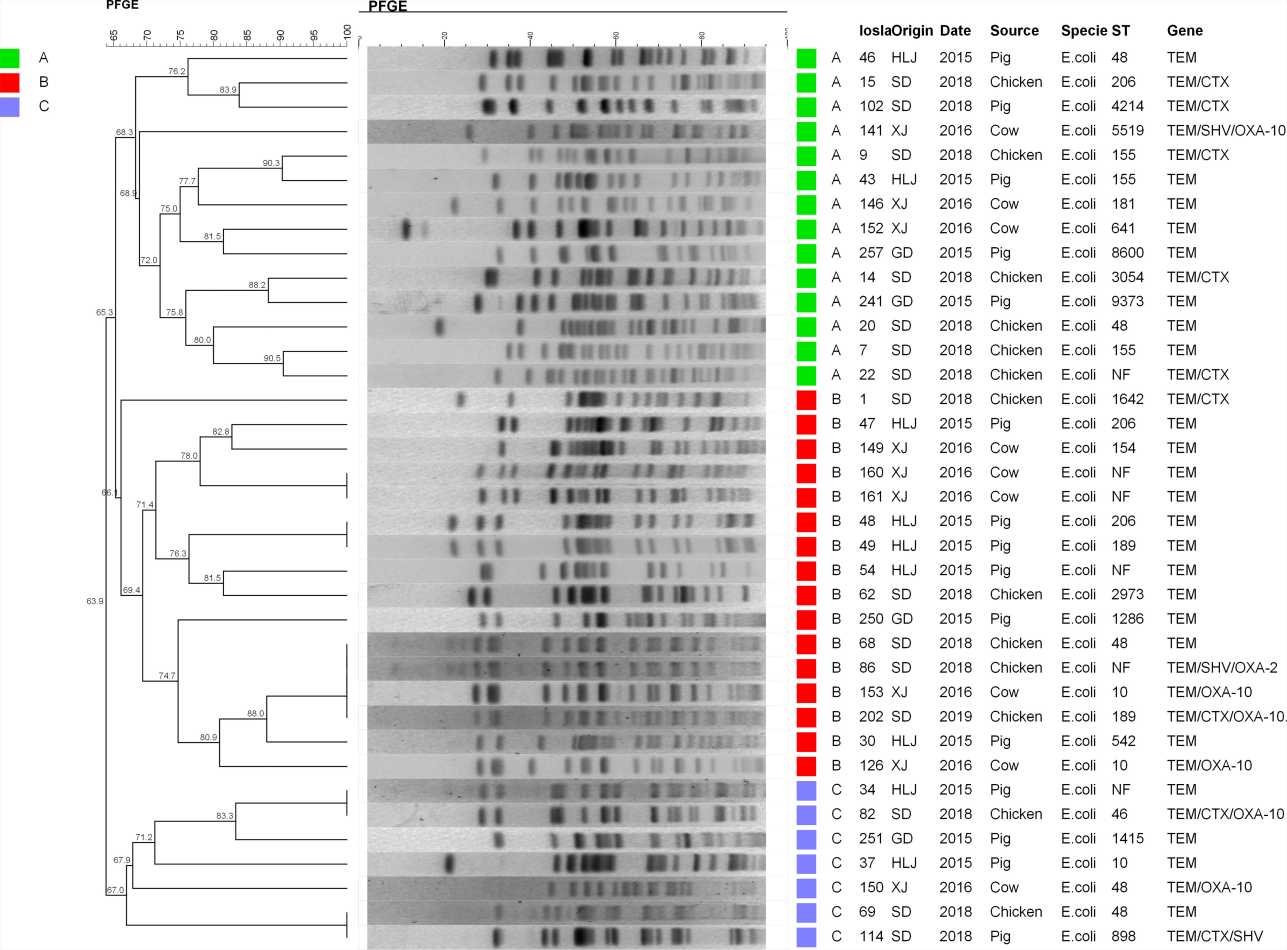
PFGE patterns of 37 ESBL-EC. All strains were mainly divided into three lineages: A (green), B (red), and C (blue). From left to right, PFGE results and phylogenetic analysis, strain number, collection place, collection time, source of strain, species, ST typing, and drug resistance genes carried are represented. HLJ, Heilongjiang; SD, Shandong; XJ, Xinjiang; GD, Guangdong.

Relationships of ESBL-EC isolates were analyzed using PFGE and MLST. Consequently, 37 isolates among 45 typical ESBL-EC were successfully identified by PFGE. These isolates could be divided into three major lineages containing 30 branches. Significantly, four different origin ESBL-EC isolates, consisting of two from chicken and one each from swine and cattle, showed the same PFGE pattern. Additionally, two small groups of chicken and swine isolates presented the same PFGE maps. Meanwhile, there were a few consistent PFGE patterns derived from the same animal species, such as w49 and w48 from swine and w161 and w160 from cattle (**Figure 3**). In general, the pattern of PFGE showed diversity but did not reveal dominant clone types.

**Figure 4:**
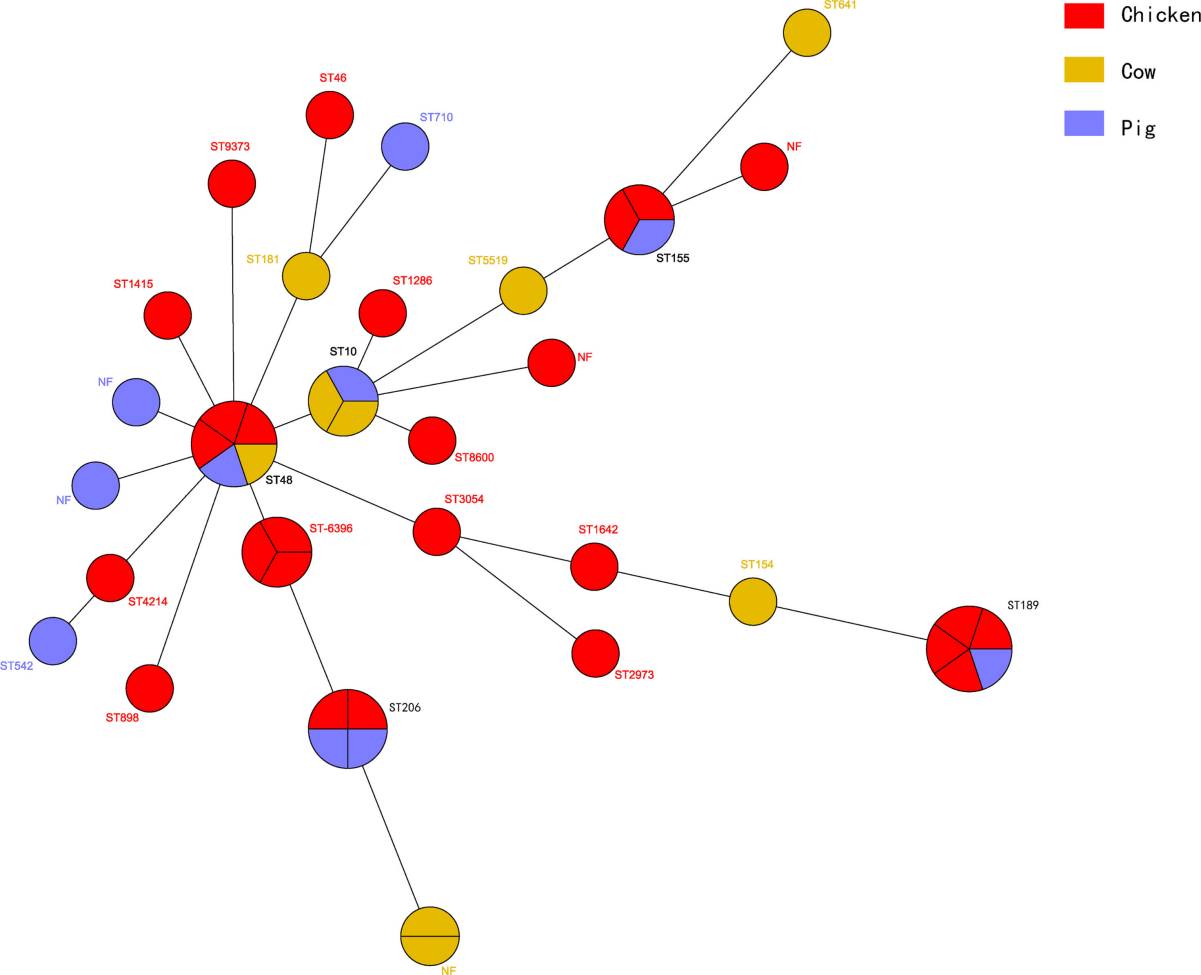
Phylogenetic analysis of STs among 45 ESBL-EC isolates. The clones from chicken (*n* = 26) were colored red, while those of cow (*n* = 9) yellow and those of pig (*n* = 10) blue.

The MLST assay was successful for 45 isolates, where 39 STs were identified and 6 isolates were not defined using the MLST database. The large clusters were ST48 (*n* = 5), ST189 (*n* = 5), ST206 (*n* = 4), ST6396 (*n* = 3), ST10 (*n* = 3), and ST155 (*n* = 3). The ST48 was present in three chicken isolates and one each from cattle and swine isolates. The five ST189 isolates consisted of one from swine and four from chicken. Interestingly, the four ST189 ESBL-EC from chicken showed coexistence of *bla*_NDM-5_ and *mcr-1*, and ST189 is the unique ST type for *mcr-1* clones in our study (**Table 2**). The STs of the remaining *bla*_NDM_-positive ESBL-EC were attributed to two STs, namely, ST6396 (*n* = 2) and ST206 (*n* = 2). All five ST48 isolates generated five diverse PFGE patterns and similar results occurred for ST189, ST206, ST10, and ST155 (**Figure 3**). Noteworthy, while three ST155 showed slightly different PFGE patterns, they still exhibited high similarity >70% (**Figures 3**, **4**). These data indicated that our ESBL-EC possessed diversity in PFGE and ST classifications and the same clonal type was commonly represented in all animal species analyzed in this study.

**Table 2:** Information for the eight *bla*_NDM_-positive *Escherichia coli* isolates identified in the current study.

Strain	Species	Sample origin	MLST type	Coexisting resistance genes	Virulence genes	Plasmids
w183	*E. coli*	Feces	ST6396	*bla*_CTX-M-14_, *bla*_TEM-1B_, *bla*_NDM-5_, *fosA*, *mph(A)*, *ph(4)-Ia*, *aac(3)-IVa*, *aph(3’)-Ia*, *sul2*	*capU*, *Is*s	IncX3, p0111, ColE10, ColRNAI
w184	*E. coli*	Feces	ST6396	*bla*_CTX-M-14_, *bla*_TEM-1B_, *bla*_NDM-5_, *aph(4)-Ia*, *aac(3)-IVa*, *aph(3’)-Ia*, *sul2*, *fosA*, *mph(A)*	*capU*, *Iss*, *gad*	IIncX3, p0111, ColE10, ColRNAII
w189	*E. coli*	Feces	ST189	*bla*_CTX-M-65_, *bla*_OXA-10_, *bla*_NDM-5_, *aph(4)-Ia*, *aac(3)-IVa*, *fosA*, *aac(6’)Ib-cr*, *mcr-1*, *aph(3’)-Ia*, *mph(A)*	*gad*	IncX3, ColE10, TrfA, Col(MG828)ColRA
w190	*E. coli*	Feces	ST206	*bla*_CTX-M-14_, *bla*_TEM-1B_, *bla*_NDM-5_, *aph(4)-Ia*, *aac(3)-IVa*, *aph(3’)-Ia*, *sul2*, *FosA*, *mph(A)*	*capU*, *iss*, *gad*	IncX3, p0111, ColE10, ColRNAI
w176	*E. coli*	Feces	ST189	*bla*_CTX-M-65_, *bla*_OXA-10_, *bla*_NDM-5_, *aph(4)-Ia*, *fosA*, *mcr-1 aac(3)-IVa*, *aph(3’)-Ia*, *aac(6’)Ib-cr*	*gad*	IncX3, ColE10, TrfA, Col(MG828), ColRNAI
w202	*E. coli*	Feces	ST189	*bla*_CTX-M-65_, *bla*_OXA-10_, *bla*_NDM-5_, *aph(4)-Ia*, *aac(3)-IVa*, *fosA*, *mcr-1*, *mph(A) aph(3’)-Ia*, *aac(6’)Ib-cr*	*gad*	IncX3, ColE10, TrfA, Col(MG828), ColRNAI
w206	*E. coli*	Feces	ST206	*bla*_OXA-10_, *bla*_TEM-1B_, *bla*_NDM-1_	NA	NA
w208	*E. coli*	Feces	ST189	*bla*_CTX-M-65_, *bla*_OXA-10_, *bla*_NDM-5_, *aph(4)-Ia*, *aac(3)-IVa*, *mcr-1 fosA*, *aph(3’)-Ia*, *aac(6’)Ib-cr*	*gad*	IncX3, ColE10, TrfA, Col(MG828), ColRNAI

NA, no available data.

### Characterization of NDM-Producing ESBL-EC

Considering the importance and significance of *bla*_NDM_, the *bla*_NDM_-positive ESBL-EC were further characterized. Transconjugation tests were employed to confirm the transferability of the *bla*_NDM_ gene and all were mobilized to strain J53 (**Table 3**). All the transconjugants displayed similar resistance profiles and conferred only a slightly lower resistance to carbapenems relative to the parental isolates. This was further evidence that *bla*_NDM_ was successfully transferred from our ESBL-EC isolates, suggesting that all *bla*_NDM_ may be located in mobile plasmids or conjugative transposons.

**Table 3:** Resistance phenotypes of eight *bla*_NDM_-positive *Escherichia coli* isolates.

Strain	MIC (µg/ml)											
	NEO	SPT	AMX	CST	TET	CN	TGC	IMP	GEN	OFL	FEP	CTX
GW176	0.5	8	256	1	1	256	0.25	256	256	0.06	32	128
GW183	0.5	8	256	1	1	256	0.25	256	256	0.06	32	128
GW184	0.5	8	256	2	1	128	0.25	256	256	0.06	32	128
GW189	0.5	8	256	2	1	256	0.25	256	256	0.06	32	128
GW190	0.5	8	256	2	1	256	0.25	256	256	0.06	32	128
GW202	0.5	8	256	2	1	256	0.25	256	256	0.06	32	128
GW206	0.5	8	256	2	1	256	0.25	256	256	0.06	32	128
GW208	0.5	8	256	2	1	256	0.25	256	256	0.06	32	128

**Figure 5:**
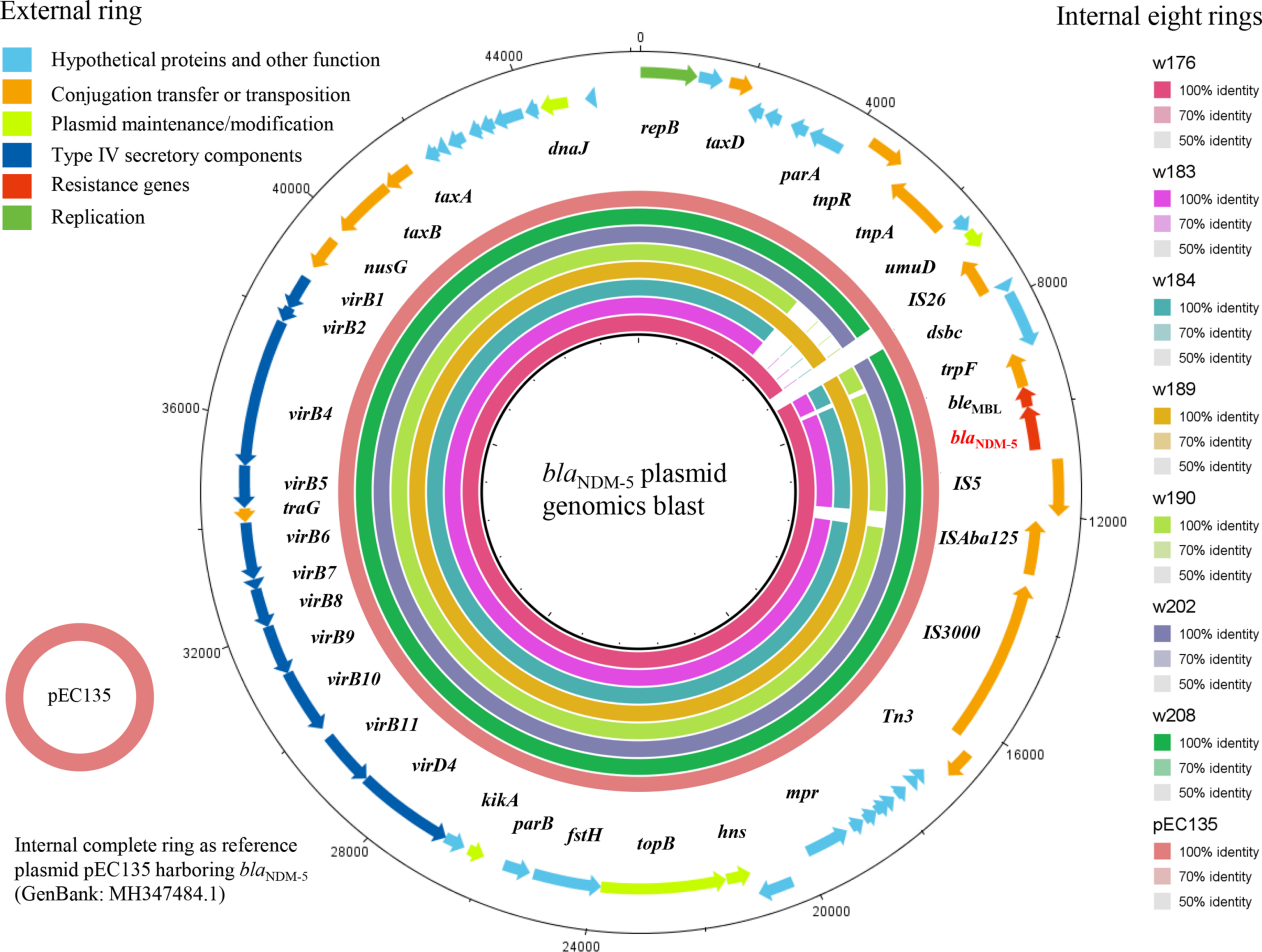
The blast and annotation of seven *bla*_NDM_-harboring plasmid genomes (constructed by BRIG). The eight small external rings represent different *bla*_NDM_-harboring plasmids and are shown in different colors, which are w176, w183, w184, w189, w190, w202, and w208 and the reference plasmid pEC135, from inside to outside. The external ring represents the annotation of plasmid pEC135 (GenBank accession no. MF347484). Genes are color-coded depending on functional annotations.

We subjected eight isolates to WGS analysis (**Table 2**), in which seven clean genomic datasets were obtained, where the clean data excluded impure and low-quality data, such as adaptor, barcode, low-quality reads, compared with the raw data, and one was excluded for the analysis due to poor data quality. Subsequently, the genomics were analyzed by CGE services, and the results are shown in **Table 2**. The results showed that these included three STs (ST189 *n* = 4, ST6396 and ST206 *n* = 2 each), where the ST of *E. coli* w206 (ST206) was performed by PCR. The coexisting resistance genes mainly included *bla*_NDM_, *bla*_CTX-M_, *bla*_OXA_, *fosA*, *mph*(A), *aph(4)-Ia*, *aac(3)-Iv*a, *aph(3’)-Ia*, *aac(6’)Ib-cr*, and *mcr-1*, notable among which was the coexistence of *bla*_NDM_ and *mcr-1*. All ST189 *bla*_NDM_ isolates contained similar ARGs, virulence genes, and plasmid types, and similar results were also observed in the two ST6396 isolates (**Table 2**). For exploring genetic environments and plasmid characteristics of *bla*_NDM-5_, all contigs harboring *bla*_NDM-5_ were extracted and annotated. Consequently, seven contigs were successfully obtained, consisting of four contigs with 45,539 bp and three contigs with 42,928 bp. These contigs shared homology >99% with many *bla*_NDM-5_ plasmid sequences in the NCBI database, such as i) pEC135 (MH347484.1) from a human *E. coli* isolate in China, ii) pJN11NDM5 (MN092230) from a human *E. coli* in China, iii) pGSH8M-2 from wastewater in Tokyo Bay, and iv) pHNAH699 (MH286952) from a chicken *E. coli* isolate in China. These plasmids were identified as IncX3 type based on typical characteristics of replication, partitioning, plasmid maintenance, transcriptional activation, and conjugation genes, as shown in **Figure 5**. Although the complete plasmid sequence was not successfully assembled, this did not prevent the seven plasmids from being identified as IncX3 type because of the typical characteristics for plasmid type harbored in these contigs. Because the remaining isolate (*E. coli* w206) genome was not successfully assembled due to low quality, it was difficult to extract a *bla*_NDM_-harboring contig and achieve plasmid typing. Given this analysis and successful transconjugation results, it can be concluded that all *bla*_NDM-5_ were located in transferable IncX3 type plasmids.

**Figure 6:**
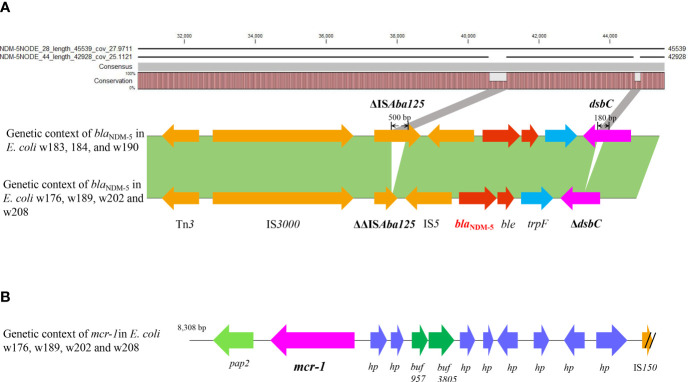
The analysis of *bla*_NDM_ and *mcr-1* genetic environments. Genetic context of *bla*_NDM-5_ (red) and the analysis of deletion fragments in ΔIS*Aba125* and *dsbC* by Blast are shown **(A)**. Genetic organization of *mcr-1* (purple) is shown in **(B)**.

To decipher the genetic context of *bla*_NDM-5_, a genomics Blast and annotation were performed on the seven contigs using pEC135 as a reference sequence (**Figure 5**). The results are exhibited in **Figure 6**, where *bla*_NDM-5_ was flanked in a typical and common genetic structure ΔIS*Aba125*-IS*5*-*bla*_NDM_-*ble*-*trpF*-*dsbC*-IS*26*, as shown in **Figure 6A**. No other resistance genes were observed in this context except for *bla*_NDM_ and *ble*_MBL_, a bleomycin resistance gene. The results of Blast showed a high identity in all seven genomes; however, a few segment gaps in three contigs (w183, 184, and w190) were observed in *umuD*, *dsbC*, and ΔIS*Aba125* regions relative to the other four genomes, which exhibit deletions in *dsbC* (180-bp deletion) and ΔIS*Aba125* (500-bp deletion). These deletions were internal to the extracted contigs rather than being generated by misassembly, suggesting that the ΔIS*Aba125* was unstable. Moreover, the genetic environments of *mcr-1* were dissected in four isolates and four genome contigs carrying the *mcr-1* were successfully extracted from them, which were small segments with sizes of 8,246 bp (w176) and 8,308 bp (w189, w202, and w208). The Blast results indicated that they shared 100% identity and the same genetic environment, which is a common sequence in the NCBI database. *mcr-1* was flanked in the upstream region by *pap2* and downstream by several hypothetical protein encoding genes, as shown in **Figure 6B**.

## Discussion

### The Increase in Omnipresent ESBL-EC Worldwide

Since the identification of the first plasmid-coded ESBL Enterobacteriaceae in the 1960s (Urbánek et al., 2007), these strains have spread globally and are a major threat to public health. In Asia, the prevalence rates of ESBL-EC are 20%~70% (Chong et al., 2018). Especially in China, the frequency of ESBL-EC is 60%~70% (Jean et al., 2016) and has been steadily increasing. Studies from 23 centers in 16 cities in China revealed that the presence rates of ESBL-EC increased from 36.1% to 68.1% among 3,074 *E. coli* over the last 10 years (*P* < 0.001) (Yang et al., 2013). A similar epidemiology has been seen in other Asian countries. Data from the Study for Monitoring Antimicrobial Resistance Trends (SMART) reported that the prevalence rate of ESBL-EC, in 2007, was 79% in India, 50.8% in Thailand, 34.4% in Vietnam, 17.8% in Hong Kong, 33.3% in South Korea and Singapore, 17.0% in the Philippines, 12.7% in Taiwan, and 55% in China (Hawser et al., 2009). By 2012, these rates had increased to 54.2% in Thailand, 28.3% in Hong Kong, 19.0% in the Philippines, 31.8% in Taiwan, and 68.8% in China (Jean et al., 2016). Recently, a global surveillance demonstrated that the rates of ESBL-EC were ~15% in Europe, ~10% in North America (Chong et al., 2018), 54%~71% (2011) in Latin America, and <15% in Africa (most countries) (Jones et al., 2013). ESBL-EC were also omnipresent, which can spread in animal food, seafood (Sanjit Singh et al., 2017), fruits (Randall et al., 2017), vegetables (Reuland et al., 2014; Randall et al., 2017), food animals (Carattoli, 2008; Day et al., 2019), wild animals (Yang et al., 2019), the environment, and even in other media, such as boots, air, insects, and transports (Müller et al., 2016; Dame-Korevaar et al., 2019). These data indicated that there is a high probability that ESBL-EC distribute to humans through food animals (Schmithausen et al., 2015; Dame-Korevaar et al., 2019). It is assessed that about 60% of human pathogens come from animals by diverse delivery media (Woolhouse, 2006). There is a serious and significantly high prevalence rate of ESBL-EC in animals relative to humans. For instance, a London study claimed that the percentage of ESBL-EC in humans was 17% (678) among 3,995 samples, but that in food animals was 65% (104) (Day et al., 2019). Chicken, cattle, and swine are the major food animals that play important roles in human health and food security, which is a global concern. Undoubtedly, the dissemination of ESBL-EC in animals is a significant risk to public safety, so effective strategies to monitor ESBL-EC prevalence should be implemented in food animals.

### The Widespread Presence of ESBL-EC for Food Animals Is Closely Associated With Veterinary Antibiotics

The emergence and prevalence of ESBL-EC are related to antibiotic usage (Ghafourian et al., 2015). However, the use of antibiotics for the treatment of infections in humans and animals is almost irreplaceable. Currently, the global annual consumption of antibiotics is between 100,000 and 200,000 tons, including human and veterinary medicine (Wang et al., 2010). Global consumption of antimicrobials in animals will increase from 63,151 in 2010 to 105,596 tons in 2030 (Van Boeckel et al., 2015), where China, USA, Brazil, India, and Germany are the largest consumers. In China, 23% of antibiotics are used for food animals at levels of 45, 148, and 172 mg/kg for cattle, chicken, and swine, respectively (Van Boeckel et al., 2015). Of all the antibiotics, the β-lactams are the most commonly used for animals, including penicillins and cephalosporins (Wei et al., 2011), both of which are associated with ESBL-EC. Accordingly, we believe, with the irreplaceable use of antibiotics in the future, the emergence and prevalence of ESBL-EC will be more serious in food animals in the absence of effective monitors.

### Differences in Resistance and Prevalence of ESBL-EC Observed in Different Food Animals

The differences in the types and amounts of veterinary antibiotics used in different animals may result in different rates of emergence and prevalence of ESBL-EC. In support of this, our study showed that resistance rates presented a significant difference in different animal origin ESBL-EC, in which the resistance to CTX, GEN, IMP, NEO, and OFL was the highest in chicken ESBL-EC, then in cattle, and the lowest in swine. A similar difference in multidrug resistance was also being observed. Meanwhile, a high prevalence of ESBL-EC was also observed, in which that of chicken (78.6%) was higher than those of cattle (70.7%) and swine (75.4%). A recent survey showed a rapid rise of chicken origin ESBL-EC from 23.8%, 25.7%, 41.2%, 44.9%, 49.6%, 50.0% to 57.0% during 2008~2014, respectively (Wu et al., 2018), but our data from chicken was even higher (78.6%). The ESBL-EC prevalence in chicken is considered to be becoming widespread and more serious than other food animals. A recent report in South Korea revealed the presence rates of ESBL-EC in chicken are the highest up to 94.1% (Song et al., 2020), followed by 69.5% in pig, while only 7.0% in cattle, where a more significant difference was observed relative to our data. The trend in prevalence rates of ESBL-EC may also reflect resistance conditions. We found that chicken origin ESBL-EC were highly resistant to all antibiotics tested in this study, compared with pig and cattle, which was similar to a previous study (Ho et al., 2011). Besides, the lower rates for pig and cattle seem to be related to antibiotic type. For example, in our results, cattle isolates exhibited high resistance rates to CTX, GEN, IMP, NEO, and OFL relative to swine, while the converse was true for AMX, SPT, TET, FEP, and COL.

### The Characteristics of *bla_ESBL_* Genes in Food Animals

The prevalence of ESBL-EC can be partly interpreted by *bla*_ESBL_ genes, which are the root occurrence of ESBL-EC. In our research, the prevalence, resistance, and *bla*_ESBL_ carriage rates of ESBL-EC present a similar trend in different food animals, where those in chicken were the highest and most serious. So, the difference of resistance in different food animals should be attributed to *bla*_ESBL_ genes. In detail, *bla*_TEM_ was present in almost all isolates, where TEM was commonly the foundation for the other β-lactamases (Pimenta et al., 2014), such as CTX-M (Bush, 2018). Over the past 20 years, TEM and SHV have been the primary β-lactamases found in ESBL-EC isolates (Zhang et al., 2014). Interestingly, the rate for SHV was low, so TEM might be the facilitator of ESBL resistance in the isolates in this study. The CTX-M type is currently the most prevalent β-lactamase globally (Zhang et al., 2014), of which CTX-M-15 and CTX-M-14 predominate in both animals and humans (Bevan et al., 2017). However, only five CTX-M-15 and four CTX-M-14 were detected, while CTX-M-55 (*n* = 69) was the predominant CTX-M prevalent subtype in food animals in our study, which agrees with the report by Pimenta et al. (2014). In China, CTX-M-55 was rare before 2009 (Zhuo et al., 2009); however, it is now the predominant ESBL and is widespread. Recently, Rao et al. described 186 CTX-M-55-expressing isolates from food animals and 47 isolates in humans (Rao et al., 2014; Zhang et al., 2014). Although CTX-M-65 is also a major CTX-M subtype in China, we only found four ESBL-EC producing CTX-M-65. As CTX-M-1 and CTX-M-14 were the dominant subtypes in other countries, CTX-M-55 spread mainly in China (Seiffert et al., 2013). This has been evidenced by recent reports in China; for instance, Fu et al. revealed 32 (84.21%) *bla*_CTX-M-55_ from 38 *bla*_CTX-M_ from humans in Shanghai (Fu et al., 2020), Zhang et al. claimed CTX-M-55 (12/27, 44.44%) was the most prevalent ESBL type from foodborne animals (Zhang et al., 2019), and Jiang et al. showed the gene *bla*_CTX-M-55_ (31/64) was the predominant *bla*_CTX-M_ subtype, followed by *bla*_CTX-M-14_ (18/64) and *bla*_CTX-M-65_ (14/64) from chicken in their study (Jiang et al., 2017).

### The Characteristics of *bla_NDM_* in ESBL-EC

More significantly, although the use of carbapenem antibiotics is banned in feed for food animals, eight NDM-producing ESBL-EC were found in 45 carbapenem-resistant isolates from chicken, consisting of seven NDM-5 and one NDM-1. As the major type of carbapenemase, NDM can impair the efficacy of almost all β-lactams (except aztreonam) and the therapeutic options are limited mostly to polymyxins and tigecycline, so acquiring additional NDM gene resistance is worrisome. Previous reports revealed that a significantly higher incidence of sepsis and bloodstream infection was caused by NDM-1 (Chatterjee et al., 2016). Since *bla*_NDM-1_ was first identified in New Delhi, India, in 2008 (Yong et al., 2009), more than 38 subtypes of NDM have been deposited in GenBank. So far, the presence of NDM has been reported in at least 55 countries and regions and in more than 60 species of bacteria (Wu et al., 2019). In our previous survey, 161 *bla*_NDM_-positive CRE (carbapenem-resistant Enterobacteriaceae) were identified among 736 food animal samples, including 84 NDM-5 (Wang et al., 2017). Currently, NDM-5 has become the predominant NDM variant among CRE from chicken (Wang et al., 2017), which is supported by our study, while NDM-1 is the predominant global NDM variant (Nordmann et al., 2011). Besides, there are many carbapenem-resistant ESBL-EC without known carbapenem resistance genes in our study, which need further exploration. Meanwhile, all transconjugation of *bla*_NDM-5_ was successfully performed, confirming the transferability of *bla*_NDM-5_ and suggesting *bla*_NDM_ location on plasmids. Subsequently, seven *bla*_NDM-5_ harboring genomic contigs were identified as IncX3 plasmid sequences. All these affirmed that the seven *bla*_NDM-5_ genes were harbored on mobile IncX3 plasmids which likely caused the rapid spread of *bla*_NDM_ in China (Ma et al., 2020). The low fitness cost of *bla*_NDM-5_-carrying IncX3 plasmid may demonstrate why it can become the predominantly transferred plasmid (Ma et al., 2020). There is no doubt that eight *bla*_NDM-5_ harboring ESBL-EC may transfer to human by the food chain, resulting in a potential threat to public health.

The genetic environment showed that all *bla*_NDM-5_ genes were flanked by the same genetic structure ΔIS*Aba125*-IS*5*-*bla*_NDM-5_-*ble*_MBL_-*trpF*-*dsbC*-IS*26*, which is a common *bla*_NDM_ genetic environment (Liu et al., 2020). Although it was regarded as being responsible for the horizontal transfer and integration of *bla*_NDM-5_ (Liu et al., 2020), further evidence is still needed to support this view. Notably, the genomes blast revealed that the *dsbC* and ΔIS*Aba125* regions of three *bla*_NDM-5_ plasmid genomes contained deletions, with 500-bp absent from ΔIS*Aba125* and 180 bp deleted in *dsbC*, compared with the other four isolates. This is a significant and powerful evidence that ΔIS*Aba125* and *dsbC* were unstable, and ever in flux, and indicated that *bla*_NDM_ as a exogenous gene was recombined into the IncX3 plasmid by insertion or transposition based on IS structure. The *bla*_NDM_ is originally from *Acinetobacter* spp. and harbored in complete transposition structure Tn*125*, including a complete IS*Aba125* on each end (Bontron et al., 2016). Subsequently, the Tn*125* composite transposon slightly changed due to each IS*Aba125* element by the part integration of IS*26*, forming an intermediate composite transposon Tn*125*-IS*26* which is adjacent to ΔIS*Aba125* and IS*26* at both termini (Weber et al., 2019). With either terminal IS*Aba125* completely replaced by IS*26*, a new composite structure ΔIS*Aba125-*IS*26* is generated, and IS*26* becomes the predominant element responsible for *bla*_NDM_ transfer (Liu et al., 2020). However, analysis of the travel history of genetic organizations of *bla*_NDM_ indicates that it currently occurs mainly in IncN/A/C type plasmids rather than IncX3 (Kopotsa et al., 2020). All these still warn us that the genetic organizations of *bla*_NDM_ are being simplified. Now, a novel evolutionary trajectory of *bla*_NDM_ genetic environment evolution is taking place in IncX3 plasmids in our study, where ΔIS*Aba125* is truncated by deletion of 500-bp sequences without IS*26* insertion and forms a shorter ΔΔIS*Aba125*, which, as far as we know, is the first report. Unexpectedly, a 180-bp segment was also lost in the *dsbC* gene downstream of the *bla*_NDM_ gene, but *dsbC* is a gene encoding an oxidoreductase rather than a transposable element. Nevertheless, it seems to suggest instability and shortening of the *bla*_NDM_ genetic locus. Accordingly, the occurrence of segment deletions in the IS*Aba125* region and even the *dsbC* gene gives us reason to hypothesize that *bla*_NDM_ loci are evolving to “pack light” to facilitate rapid and stable transfer.

### Diversity in ST and PFGE

ST and PFGE diversity were observed in the major ESBL-EC in our study, and shared STs and PFGE patterns were exhibited. The presence of similar ST and PFGE types indicated that it is possible for ESBL-EC to transfer among the different food animals. Presently, ST131 has become a major ST across the globe for ESBL-EC (Marie-Hélène et al., 2014), especially those carrying *bla*_CTX-M_. There is widespread ST131 in both humans and animals (Belas et al., 2019), especially in chicken, swine, and cattle (Duggett et al., 2021). However, there were no ST131 isolates identified in our study, while a distribution of types was noted including ST48 (*n* = 5), ST189 (*n* = 5), ST206 (*n* = 4), ST6396 (*n* = 3), ST10 (*n* = 3), and ST155 (*n* = 3). The same five ST189 isolates included one from swine and four from chicken. Notably, four ST189 from chicken harbored the same resistance genes, namely, *bla*_CTX-M-65_, *bla*_OXA-10_, *bla*_NDM-5_, and *mcr-1.* ST189 *E. coli* mainly spread in poultry and is thought to have spread from food animals to human (Kim et al., 2015; Hasan et al., 2016). Previously, ST189 ESBL-EC was reported to be associated with *bla*_CTX-M_ (Hasan et al., 2016), in agreement with our study. Recently, a series of reports revealed *mcr-1* was found in ST189 *E. coli* from food animals and human (Zając et al., 2019; Yamaguchi et al., 2020), suggesting that ST189 ESBL-EC have become the reservoirs, even and preferred clones of *mcr* genes, as we also found. However, *bla*_NDM_ or variants were hardly reported in ST189 *E. coli*, indicating that our study revealed novel findings on *bla*_NDM_ in ST189 ESBL-EC. The occurrence of ST206 *E. coli* was mainly in poultry farms, which often carried the *bla*_ESBLs_. Recently, Ayeni et al. reported that 35 (68.6%) ST206 *E. coli* harbored *bla*_ESBLs_ in poultry in Nigeria (Ayeni et al., 2020), and CTX-M-27 was deemed to be almost exclusively found in ST206 isolates (Ayeni et al., 2020). ST206 *E. coli* was often identified in carbapenemase-producing CRE or colistin-resistant isolates from animal, environment, and human, often harboring *bla*_NDM_ or *mcr-*1, sometimes even with coexisting *bla*_NDM_ and/or *mcr-*1 genes. ST206 *E. coli* harboring *bla*_NDM_ or/and *mcr-*1 have been found in acute diarrhea patients or outpatients (Zheng et al., 2018), retail vegetables (Luo et al., 2017), pig, and water samples (Li et al., 2019). This was also observed in our study, where two ST206 *E. coli* were derived from food animals. All these suggested that *mcr* and/or *bla*_NDM_-carrying ST206 *E. coli* can transfer to human by the food production chain (Kawamura et al., 2017; Li et al., 2019). On the other hand, ST48 ESBL-EC was present in three animal species, but these displayed different PFGE patterns, suggesting that they were different strains. ST48 was reported in human and animal isolates as associated with ESBL lactamases, especially the CTX-M type (Chen et al., 2019; Nüesch-Inderbinen et al., 2020), but we did not find the latter in any of our ST48 isolates. ST10 is considered to be the second most prevalent clone after ST131 in ESBL-EC worldwide and is mainly spread among livestock and poultry (Aibinu et al., 2012), such as swine, chicken, and cattle (Pietsch et al., 2017). This was supported by our research, as two ST10 ESBL-EC were from cattle and the other one was from swine. ST10 with *bla*_CTX-M_ was not found in our study as the three ST10 ESBL-EC did not carry this gene. Similarly, ST155 was previously detected in CTX-M-1-producing *E. coli* strains, while no *bla*_CTX-M_ was observed in our three ST155 ESBL-EC. Currently, the major prevalent source of ST155 ESBL-EC is chicken and its environments. Like ST155, ST6396 is also a niche prevalent clone, but detected in human and animals (Zhao et al., 2021). In general, shared ST clones will become more frequent in food animals. In other words, the diversity of ESBL-EC would be more common in any food animal in the future.

## Conclusions

In our study, ESBL-EC showed high prevalence and diverse isolate lineages among chicken, swine, and cattle, together with a significant difference in prevalence rates of *bla*_ESBL_ genes and resistance determinants. All these indicate that farm animals have become reservoirs of ESBL-EC and facilitators of *bla*_ESBL_ gene transfers and which can also show genetic environment change. Meanwhile, the coexistence of *bla*_NDM_ and/or *mcr* has emerged in ESBL-EC among food animals, posing further threats to human health. Accordingly, it is time for more effective measures to monitor the prevalence of ESBL-EC in different food animals, reducing the risk of transmission to humans.

## Data Availability Statement

The datasets presented in this study can be found in online repositories. The names of the repository/repositories and accession number(s) can be found below: BioProject accession no. PRJNA784017 including JAJNDN000000000, JAJNDO000000000, JAJNDP000000000, JAJNDQ000000000, JAJNDR000000000, 572 JAJNDS000000000, JAJNDT000000000 (https://www.ncbi.nlm.nih.gov/genbank/).

## Ethics Statement

The animal study was reviewed and approved by Qingdao Agricultural University Animal Experiment Committee. Written informed consent was obtained from the owners for the participation of their animals in this study.

## Author Contributions

ZH and ZL are responsible for the study design. KW, ZL, LX, LQ, LZ, CG, and XL assisted in the data collection. KW and ZL interpreted the data. ZL and YZ completed the written report. All authors contributed to the article and approved the submitted version.

## Funding

This work was supported by the National Key Research and Development Plan (No. 2016YFD0501007); National Natural Science Foundation of China (No. 32172911); 100 foreign experts and teams plan (No. WST2017010); Shandong Provincial Natural Science Foundation, China (No. ZR2020QC199); and China Postdoctoral Science Foundation (No. 2021M701105).

## Conflict of Interest

Author YZ was employed by Shandong New Hope Liuhe Group Ltd. Author KW was employed by company Autobio Labtec Instruments Co., Ltd.

The remaining authors declare that the research was conducted in the absence of any commercial or financial relationships that could be construed as a potential conflict of interest.

## Publisher’s Note

All claims expressed in this article are solely those of the authors and do not necessarily represent those of their affiliated organizations, or those of the publisher, the editors and the reviewers. Any product that may be evaluated in this article, or claim that may be made by its manufacturer, is not guaranteed or endorsed by the publisher.
